# Navigating fragmented landscapes: Canada lynx brave poor quality habitats while traveling

**DOI:** 10.1002/ece3.4605

**Published:** 2018-10-26

**Authors:** Carmen Vanbianchi, William L. Gaines, Melanie A. Murphy, Karen E. Hodges

**Affiliations:** ^1^ Biology Department University of British Columbia Okanagan Kelowna British Columbia Canada; ^2^ Washington Conservation Science Institute Leavenworth Washington; ^3^ Department of Ecosystem Science and Management, Program in Ecology University of Wyoming Laramie Wyoming

**Keywords:** habitat quality, landscape permeability, least cost paths, *Lynx canadensis*, North Cascades, resistance maps, Washington, wildfire

## Abstract

Anthropogenic and natural habitat fragmentation inhibit movement of animals through landscapes. An important challenge for connectivity conservation is determining which conditions facilitate or limit movements, so that these areas can be prioritized for protection or restoration. We examine Canada lynx Lynx canadensis habitat connectivity in the fragmented North Cascade Mountains of Washington, as an example of a highly mobile species that is specialized both on prey and in habitat needs. We identify lynx Habitat Concentration Areas based on Core Habitat Models, parameterize resistance surfaces from our Matrix Habitat Model, and develop linkages of habitat lynx use to move between patches of high quality habitat. We identify a number of linkages for lynx comprised of habitat conditions that differed from high quality core patches identified from our habitat modeling. Radio‐locations from lynx confirm lower‐quality habitats of low resistance to movement were used by traveling lynx. Our results thus suggest traveling lynx do indeed use a much broader range of habitats than do lynx moving within core areas. For lynx in the North Cascades, our results show that maintaining connectivity will require preserving habitats and linkages that would previously have been deemed unsuitable for lynx. Maintaining connectivity for lynx is particularly important given the many recent large wildfires in this region that have reduced the number of mature forest stands that form prime habitat for lynx. Policy implications. Our results strongly suggest that habitat connectivity models should be based on empirical information of animal location data and focused on matrix habitat analysis. Traveling predators use a wide suite of habitats, resulting in more and broader linkage zones that should inform conservation efforts. Failure to identify these areas of functional connectivity could result in the oversight of usable linkage zones, leaving them without protection and vulnerable to degradation.

## 1 INTRODUCTION

Healthy ecosystem function relies in large part on movements by organisms: mammals travel to find food, fish migrate from oceans to streams to spawn, and seeds disperse across the landscape. These movements occur at different spatial and temporal scales (Crooks & Sanjayan, 2006). Importantly, as humans increasingly alter the planet, the movements of wildlife are inhibited by development, deforestation, roads, and a variety of other human‐induced features. Habitat loss and fragmentation have become top factors in species declines around the world (Brooks et al., 2002; Ewers & Didham, 2006; Wilcove, Rothstein, Dubow, Phillips, & Losos, 1998). Connectivity conservation has emerged as an important strategy for mitigating the effects of fragmentation (Crooks & Sanjayan, 2006; Ewers & Didham, 2006).

Habitat fragmentation and the associated loss of connectivity have many negative consequences (Keinath et al., 2016). Habitat fragmentation can impede animals dispersing to a new home range and obstruct the movement of individuals seeking mates or resources (Fischer & Lindenmayer, 2007; Wilcox & Murphy, 1985). Fragmentation can also separate populations (Hanski, 1998), inducing genetic isolation and inbreeding depression (Frankham, 2006). Finally, as climate change and other human impacts cause habitat degradation and loss, populations may need to shift their ranges to escape poor conditions, relying on connected landscapes to facilitate range shifts (Chen, Hill, Ohlemüller, Roy, & Thomas, 2011; Lenoir & Svenning, 2015; Parmesan, 2006).

Structural connectivity models focus on how well particular habitats are linked, rather than basing models on documented movements of focal species. Structural connectivity is based on connecting physical attributes of a landscape (Tischendorf & Fahrig, 2000), often using a binary description in which islands of habitat are surrounded by a uniformly inhospitable matrix (Wiens, 2006). However, this approach to connectivity fails to consider the many cases for which the matrix is not an entirely hostile environment (Chetkiewicz, St. Clair, & Boyce, 2006; Prugh, Hodges, Sinclair, & Brashares, 2008); many landscapes are better characterized as containing a spectrum of habitat quality.

Functional connectivity considers an animal's behavioral responses to the various landscape features, recognizing that presumed non‐habitat may be used for travel (Tischendorf & Fahrig, 2000). Thus, a landscape that appears structurally unconnected may in fact be connected if the intervening matrix is permeable for traveling animals. Similarly, a landscape that appears to be structurally connected may be functionally unconnected if the corridor is too narrow to buffer an animal from surrounding inhospitable habitats, or if the corridor is longer than the animal's maximum dispersal distance (Beier, Majka, & Spencer, 2008; Taylor, Fahrig, & With, 2006; Tischendorf & Fahrig, 2000). Furthermore, a functionally connected landscape may not be based on distinct corridors of quality habitat, but rather the overall permeability of matrix habitats. Because functional connectivity incorporates animal behavior and habitat use, this definition of connectivity is a more fruitful approach for conservation planning when salient data are available (Chetkiewicz et al., 2006; Tischendorf & Fahrig, 2000).

Identifying functional connectivity requires researchers to have a thorough understanding of the focal species’ behavioral responses to landscape features. Modelers typically assign numeric values to landscape features that influence the movements of the focal species, such as topography, habitat types, or human disturbances (Beier et al., 2008), with high resistance values indicating that a landscape feature is either highly avoided or results in a loss of fitness or low survival for animals passing through the landscape feature (Zeller, McGarigal, & Whiteley, 2012). Resource selection models based on locations of animals and the habitat features in a region (Chetkiewicz & Boyce, 2009; Vanbianchi, 2015; Vanbianchi, Murphy, & Hodges, 2017; Vanbianchi, Murphy, Pither, Gaines, & Hodges, 2017) thus provide an empirical foundation for assigning resistance values to landscape maps, upon which connectivity models should be based.

Resource selection models are often based on locations pooled from animals in their home ranges, thus revealing general habitat selection. But because animals often select different habitats for different activities (Roever, Beyer, Chase, & Aarde, 2014), using resource selection models across these varying behaviors and habitats becomes problematic for connectivity modeling. Specifically, animals may use the most resource‐rich habitats (“core” habitat hereafter) for daily activities such as foraging or resting, but may use additional habitats for traveling across home ranges and especially when dispersing outside home ranges (Roever et al., 2014). If researchers fail to recognize that an animal uses a wider range of habitats for traveling than for core habitats, then managers could underestimate connectivity, misdirect management efforts, or even damage existing areas of genuine connectivity that are thought to be unsuitable. Thus, models based on data not only from core habitats but from animals crossing lower quality habitat (“matrix” habitat hereafter) are likely to provide more accurate resistance values for modeling functional habitat linkages. Indeed, several recent studies have found that connectivity models were more informative when using resistance surfaces based on habitat selection analysis linked to movement behavior outside an animal's core habitat (Blazquez‐Cabrera et al., 2016; Keeley, Beier, & Gagnon, 2016; Keeley, Beier, Keeley, & Fagan, 2017; Trainor, Walters, Morris, Sexton, & Moody, 2013).

In the western United States, many forest habitats are naturally and anthropogenically fragmented. Sub‐boreal forests are limited to high elevations, such that topography itself fragments habitat (Agee, 2000). Climate change is further shrinking the range of sub‐boreal forests northward and upward in elevation (Franco et al., 2006; Soja et al., 2007), and may affect peripheral populations of animals sooner than those in the central part of their range (Anderson et al., 2009). In addition, climate change is increasing the frequency, size, and intensity of wildfires, further fragmenting forest habitats (Fauria & Johnson, 2007; Littell et al., 2010; Soja et al., 2007). Finally, human disturbances such as roads, development, and timber harvest fragment these habitats (Buskirk, 2000; Koehler et al., 2008).

Canada lynx *Lynx canadensis* Kerr provide an interesting case study for functional connectivity mapping because structural connectivity does not adequately describe the complex movements of lynx through the landscape. Lynx are specialized predators on snowshoe hares *Lepus americanus* Erxleben, are wide‐ranging (dispersal distances up to 100s of km), yet have suffered from range retraction and population declines in the southern edge of their range that may be tied to habitat loss and fragmentation (Buskirk, 2000; Hornseth et al., 2014; McKelvey, Aubry, & Ortega, 2000). Lynx are federally listed as Threatened (USFWS, 2000) and are state‐listed as Endangered in Washington (Lewis, 2016). Retaining southern lynx populations will require landscapes that support regular movement of lynx among remnant patches of high quality habitat within their home ranges and more broadly across lynx range. The high mobility of lynx suggests they can use a wide variety of habitats while traveling or dispersing, but their reliance on snowshoe hares as prey and their strong affinity to snowy boreal forest habitats suggests such patches must be connected if lynx are to be kept in landscapes that historically supported them.

Understanding functional connectivity for species of conservation concern such as lynx is a critical need, especially since wildfires continually and increasingly repattern their forested habitat. To address this need, we develop robust predictions of habitat connectivity and linkage zones for lynx in their southwestern range edge, the North Cascade Mountains of Washington, USA, (U.S. Fish & Wildlife Service, 2000). Our specific objectives were to model lynx habitat concentration areas based on our core habitat resource selection model (Core Habitat Model, hereafter), to develop a resistance surface for lynx based on our matrix habitat resource selection model (Matrix Habitat Model, hereafter), and to then combine maps of habitat concentration and resistance to model connectivity of North Cascades lynx habitat. Our habitat models were constructed using 20,564 GPS locations from 17 lynx and both the Core Habitat Model and Matrix Habitat Models included explicit examination of lynx use of recently burned areas (see also Vanbianchi, Murphy, & Hodges, 2017). Our results point to clear areas most likely to support lynx movement between the remaining patches of high quality forest for lynx in the North Cascades.

## 2 MATERIALS AND METHODS

**Figure 1:**
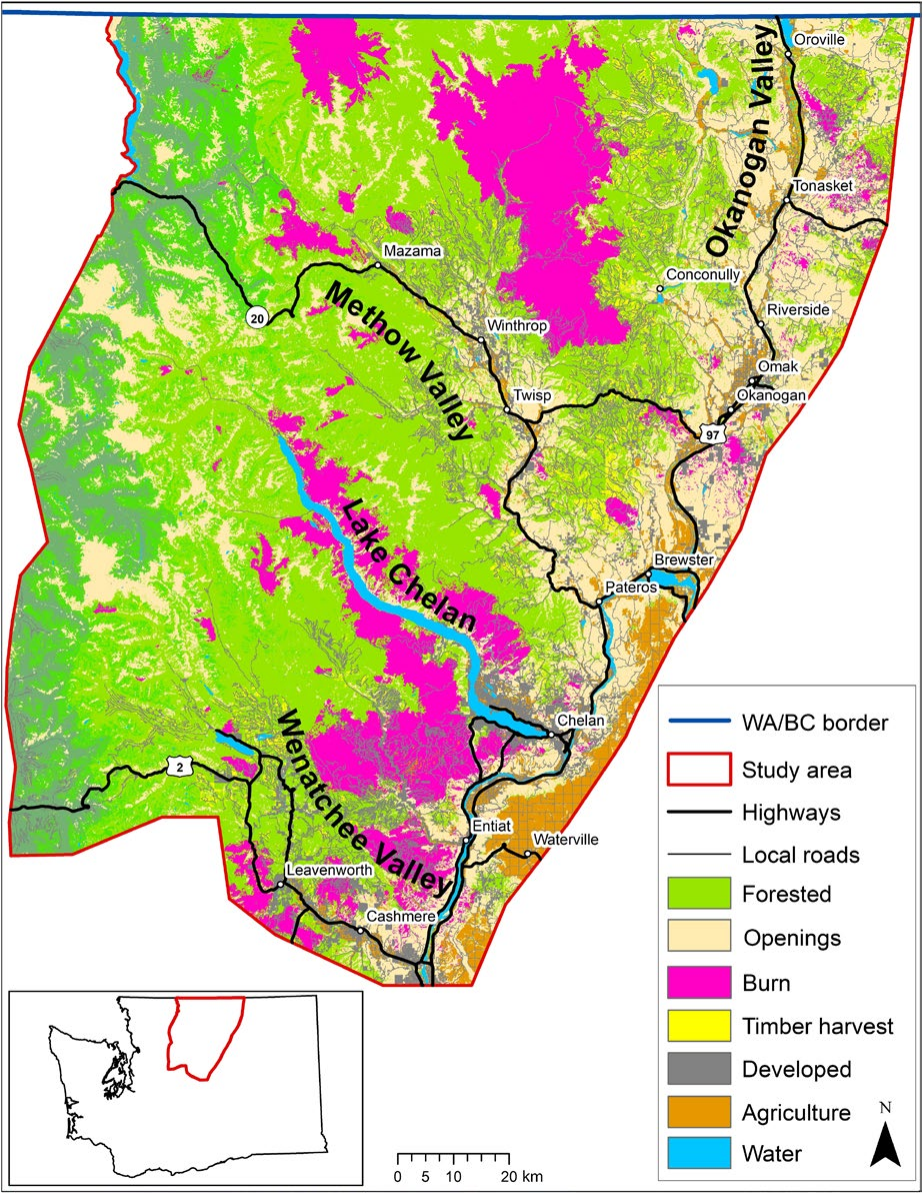
The North Cascades study area of northcentral Washington across which lynx habitat connectivity was modeled. The Black Pine Basin and Loomis focal areas where our Core Habitat and Matrix Habitat models were developed are just north of Mazama and Winthrop

We modeled lynx functional connectivity throughout the North Cascade Mountains of northcentral Washington. The North Cascades study area included 20,260 km^2^ from the British Columbia‐Washington border southward to 10 km south of Highway 2, and from 25 km west of the Cascade crest to 15 km east of Highway 97 (Figure 1). The North Cascades study area includes all of the Okanogan Lynx Management Zones designated by the Washington Department of Fish and Wildlife (Stinson, 2001). Most of the study area (78%) is public land with private property concentrated in low‐elevation areas such as the Okanogan and Methow Valleys; developed private properties comprise 4% of the study area (Vanbianchi, 2015).

The study area is mountainous, with elevations ranging from 188 to 3,214 m, and 60% of the area above 1,000 m. Forests grow at higher elevations and on north‐facing slopes at lower elevations. Open shrublands dominate low‐elevation areas and south‐facing slopes. During 2006–2013, the study area was approximately 50% forested, but only 14% of the study area was comprised of the sub‐boreal forests lynx select in this region (Vanbianchi, 2015). Open areas (shrubs, alpine, grassland) covered 30% of the study area. Disturbances (since 1985) caused by wildfires or timber harvest cover 16% of the study area. The largest disturbance was the 70,644 ha Tripod Fire, which burned much of Washington's known lynx habitat in 2006 (Agee, 2000; Koehler et al., 2008; Stinson, 2001). Nearly, 22,000 km of roads exist on the study area, ranging from closed forest roads to major highways. Snowshoe hares occur with moderate densities in areas with adequate forest cover (Lewis, Hodges, Koehler, & Mills, 2011). In 2017, after we developed these models, the Diamond Creek Fire (51,648 ha) burned 35,445 ha of the “core habitat” within the northern part of the study area.

To model functional connectivity for lynx throughout the North Cascades, we first developed two Random Forest models of habitat use by lynx (Vanbianchi, 2015; Vanbianchi, Murphy, & Hodges, 2017; Vanbianchi, Murphy, Pither, et al., 2017): the Core Habitat Model and the Matrix Habitat Model. These models identified the habitat variables important for defining core and matrix habitat for lynx in the North Cascades and were based on location data obtained from lynx trapped and fitted with Global Positioning System (GPS) telemetry collars in the Okanagan‐Wenatchee National Forest and the Loomis State Forest from 2006 to 2012. Trapping took place collaboratively by the Washington Department of Fish and Wildlife, Washington Department of Natural Resources, U.S. Forest Service, U.S. Bureau of Land Management, and the U.S. Fish and Wildlife Service (J. Rohrer, personal communication).

For the habitat models, we used lynx locations from within their home ranges. Lynx home ranges were clustered in two separate areas that we delineated as focal areas within the North Cascades study area: the Black Pine Basin and Loomis focal areas. We used 4,113 lynx locations compared to an equal number of random available locations within the Black Pine Basin and Loomis focal areas to develop our Core Habitat Model. Random locations were identified from within each focal area of lynx locations, buffered by 766 m, the average distance between 4 hr fixes from collared lynx. This model depicted the habitat where probability of lynx use was high and that was presumably used for hunting and resting. Because core habitat in the North Cascades is fragmented even within a lynx’ home range, we were then able to develop our Matrix Habitat Model by using only lynx locations from between the previously modeled core habitat patches in matrix areas. We defined matrix as those habitats predicted by the Core Habitat Model as having <45% probability of use. Using this probability threshold insured we were exploring areas that lynx are unlikely to choose for hunting or denning. Although we could have used a lower threshold (e.g., <30%) to signal much lower habitat desirability, we wanted to retain enough data points for a reasonable model. By comparing 404 lynx locations from within matrix areas, to an equal number of random available locations within matrix areas, our model elucidated lynx habitat selection at the lesser used, low end of the habitat quality spectrum.

We developed the Core and Matrix habitat models using Random Forest (Breiman, 2001) implemented in R version 3.2.1 (R Core Team, 2014) using rfUtilities (Evans & Cushman, 2009; Evans & Murphy, 2014; Evans, Murphy, Holden, & Cushman, 2011) to compare the habitat variables present at lynx GPS locations and random available locations (Vanbianchi, 2015; Vanbianchi, Murphy, & Hodges, 2017; Vanbianchi, Murphy, Pither, et al., 2017). Habitat variables were depicted with raster data layers developed in ArcGIS 10.1 (ESRI, 2012). Habitat variables used represented land cover types, topography, climate, forest structure, patch metrics, and disturbances. Our habitat variables included several fire‐related elements allowing us to discover the effects of burn age and severity, the importance of fire skips, and distance to the edge of a burn (Vanbianchi, 2015; Vanbianchi, Murphy, Pither, et al., 2017). We created continuous representations of each habitat variable using 30 m^2^ pixels projected into the 1983 North American Datum Albers coordinate system (See Vanbianchi, 2015 and Vanbianchi, Murphy, & Hodges, 2017 for a description of layer development and data sources).

### 2.1 Identification of habitat concentration areas

To model connectivity in the North Cascades, we first identified Habitat Concentration Areas (Singleton, Gaines, & Lehmkuhl, 2002; WWHCWG, 2010). We created a habitat quality raster by extrapolating the results of the Core Habitat Model beyond the Black Pine Basin and Loomis focal areas across the larger North Cascades study area (Vanbianchi, 2015; Vanbianchi, Murphy, & Hodges, 2017; Vanbianchi, Murphy, Pither, et al., 2017). This raster depicted the probability of lynx use for each pixel, which we equated with underlying habitat quality. These values were scaled from 1 (poor habitat) to 10 (good habitat).

Seventeen variables were used in the Core Habitat Model as important predictors of lynx occurrence (Vanbianchi, 2015; Vanbianchi, Murphy, & Hodges, 2017; Vanbianchi, Murphy, Pither, et al., 2017). Each variable was assessed at broad and fine scales (27 × 27 pixels, 3 × 3 pixels). We chose these scales to reflect both the immediate neighborhood around a lynx (3 × 3 pixels) and what we hypothesized as the largest‐scale perceived by a lynx operating within its home range (27 × 27 pixels).

As we detailed elsewhere (Vanbianchi, 2015; Vanbianchi, Murphy, & Hodges, 2017; Vanbianchi, Murphy, Pither, et al., 2017), lynx selected areas with sub‐boreal “spruce‐fir” forests dominated by lodgepole pine (*Pinus contorta* Douglas) or Engelmann spruce (*Picea engelmannii* Parry) and subalpine fir (*Abies lasiocarpa* (Hook.) Nutt.), while dry forests, characterized by Douglas fir (*Pseudotsuga menziesii* (Mirb.) Franco) and Ponderosa pine (*Pinus ponderosa* Douglas) were selected against. Lynx also selected “mixed forests” transitioning between sub‐boreal types and dry forests dominated by Douglas fir and intermixed with sub‐boreal species. Lynx avoided grasslands, shrub‐steppe, old thins, areas recently burned at high severity, areas within a burn perimeter, steep slopes, and areas with sparse canopy cover. Climate variables were also important. Lynx selected for areas with greater moisture accumulations as depicted by the Compound Topographic Index, a measure of moisture accumulation based on slope and upslope area (Gessler, Moore, McKenzie, & Ryan, 1995; Moore, Gessler, Nielsen, & Petersen, 1993). Lynx selected for cooler, moister slopes as depicted by the Heat Load Index, which incorporates both aspect and slope (McCune & Keon, 2002). Finally, lynx selected areas with greater amounts of growing season precipitation. In all cases, variables describing lynx habitat use were more important at a large scale, although three variables were important at both scales (new high‐severity burn, slope, and canopy cover).

Next, we added six landscape variables that are hypothesized to impact lynx and were present on the North Cascades study area, but that were not present in the Black Pine Basin or Loomis focal areas and hence, were not included in our Core or Matrix Habitat Models. Values for these variables were based on expert opinion (three of the authors and three other experts familiar with lynx and the region). These experts were consulted in February 2015. A value of 0 represented no impact on lynx habitat, 10 represented a major negative impact, and negative numbers represented a positive impact on lynx habitat (Table 1). To adjust the habitat quality raster, we subtracted the average of these assigned values from affected pixels. For example, in areas within 50 m of road, the habitat value in the habitat quality raster was lowered by 4. Although Baigas, Squires, Olson, Ivan, and Roberts (2017) found that lynx on Colorado did not select against highways, roads do present the danger of vehicle strikes to lynx and thus increase resistance to successful lynx movement.

**Table 1:** Landscape variables used in the connectivity modeling that were developed from expert opinion from six people

Habitat variable	Decrease in quality of core habitat	Decrease in quality of travel habitat
Distance to developed area (m)[Link]		
0	8	4
1–50	6	2
50–100	3	1
100–250	1	0
Distance to highways (m)		
0	7	3
1–50	4	1
50–100	2	0
100–250	0	0
Cover categories[Link]		
West‐side sub‐boreal forest	2	−1[Link]
West‐side wet forest	6	1
Agriculture	7	5
Water	6	4

Notes

These variables were not included in the telemetry‐based habitat modeling, but were thought to be important to lynx in the more extensive landscape used for connectivity modeling. Experts were asked to rank each item from 0 (no impact) to 10 (major negative impact); negative values indicate a benefit to lynx habitat; values given here were the average from the six opinions. For roads and developed areas, experts judged there were no impacts for distances of 250–500 m, 500–1,000 m or above 1,000 m.

Tax parcels with residential or commercial development.

The four cover categories were assigned values because the habitat models did not include those cover types and we needed values for the connectivity maps. West‐side sub‐boreal forest is wetter than east‐side sub‐boreal forests. West‐side wet forest is lower elevation than west‐side sub‐boreal forest zone. “Water” includes large lakes and rivers.

The presence of sub‐boreal forest on the west side is thought to slightly improve the habitat quality for a traveling lynx.

During the next step of identifying Habitat Concentration Areas within the North Cascades, we used the R program package adehabitatHR (Calenge, 2006) to estimate home ranges (95% minimum convex polygons) for each radio‐collared lynx that localized in the Black Pine Basin or Loomis areas and provided at least six months of data. Excluding Male 339, who did not have a well‐localized home range, the average home range was 88 km^2^ (Table 2). We used each home range polygon and the adjusted habitat quality raster to calculate the average habitat value within each lynx home range. Male 336 was excluded from this analysis since his home range straddled the Washington/British Columbia border and was thus partly outside the study area and beyond the limit of the habitat quality raster.

**Table 2:** Minimum convex polygon (MCP) home range estimates for lynx, derived from GPS location data collected in the North Cascades

Lynx ID	95% MCP home range in km^2^	Average habitat value per pixel	Standard deviation
Male 339	674	5.0	2.7
Male 327	231	3.8	1.9
Male 311	127	5.9	2.1
Male 338	116	7.6	2.3
Male 346	98	7.4	1.8
Male 347	78	7.4	2.0
Male 309	75	8.0	1.8
Male 329	73	6.0	1.8
Male 336	36	–	–
Male 308	36	8.9	1.1
Male 348	19	7.9	1.9
Female 340	131	6.1	1.9
Female 330	67	6.8	1.5
Female 349	61	8.6	1.4

The average habitat value per pixel was calculated within each lynx’ home range, excluding lynx 336 since a large portion of his home range fell beyond the limit of the habitat quality raster. Lower numbers indicate poorer average habitat.

Our final step in developing Habitat Concentration Areas was a moving window analysis across the habitat quality raster (Core Mapper in ArcGIS; Shirk & McRae, 2013). We used an 88 km^2^ moving window to reflect the average home range size of lynx (Table 2). For each pixel, the moving window calculated the average habitat value of pixels surrounding it. We then extracted all pixels with an average neighborhood value >3.8, the lowest average habitat value used by any of the GPS‐collared lynx. We used the lowest average habitat value because it resulted in an ample distribution of Habitat Concentration Areas that allowed us to model habitat linkages between them; using a higher value would have meant smaller and more fragmented Habitat Concentration Areas. We split the largest Habitat Concentration Area in two, creating a northern and southern area since our Least Cost Path analysis would only create a single path per Habitat Concentration Area. By splitting this Habitat Concentration Area, the Least Cost Path analysis would locate a path on both the northern and southern halves rather than a single path for the entire Habitat Concentration Area thus increasing the number and distribution of Least Cost Paths over such a large area.

### 2.2 Creating the resistance surface

To create a resistance surface for modeling habitat linkages, we applied the results of the Matrix Habitat Model (Vanbianchi, 2015; Vanbianchi, Murphy, Pither, et al., 2017), which identified the features lynx select while crossing through low‐quality habitat. For this model, we used locations with a <45% probability of use by lynx. We extrapolated these results beyond our Black Pine Basin and Loomis focal areas to throughout the broader North Cascades study area and scaled the raster so that a value of 1 represented areas of no resistance to movement, and 10 represented areas of high resistance to movement. The Matrix Habitat Model identified 20 variables that predicted how lynx select habitat while traveling through matrix areas (Vanbianchi, Murphy, Pither, et al., 2017). Lynx selected matrix habitats that included a wider range of habitat conditions compared to core habitats. For example, lynx were more tolerant of new, high‐severity burns, namely the Tripod Burn, while using matrix habitats. Lynx preferred to use areas of the Tripod Burn closer to the edge and large‐scale areas if low‐severity burns, fire skips, or old burns were also within the large‐scale area. For traveling lynx, deciduous forest, new clearcuts, and the compound topographic index (at a small scale) were also minor predictors of habitat use. As with the habitat quality raster, we adjusted the resistance surface created by the matrix habitat raster by using expert opinion to incorporate important habitat variables missing from the Matrix Habitat Model but present within the greater North Cascades landscape (Table 1).

### 2.3 Modeling connectivity

To identify Least Cost Paths that linked the Habitat Concentrations Areas, we conducted a connectivity analysis in linkage Mapper 1.0 (McRae & Kavanagh, 2011), thus modeling connectivity for lynx across the North Cascades. First, we performed a cost‐weighted analysis by calculating the cost of moving from any pixel on the landscape to a selected Habitat Concentration Area, the cost of a pixel being its resistance value times the width of the pixel. This step produced an individual cost‐weighted distance raster for each Habitat Concentration Area.

We determined which Habitat Concentration Areas were adjacent to each other by using Linkage Mapper to calculate both Euclidean distance and cost‐weighted distance. Each individual cost‐weighted distance raster was then combined with those of adjacent Habitat Concentration Areas by retaining the lowest value for each pixel. By combining individual cost‐weighted distance rasters in this way, we produced a map displaying the weighted cost that would be accrued traveling from each pixel on the landscape to the nearest Habitat Concentration Area (McRae & Kavanagh, 2011).

We then used Linkage Mapper to calculate the pixel‐wide Least Cost Path between each adjacent Habitat Concentration Area. Linkages were then mapped between Habitat Concentration Areas by adding together the pixel values of individual cost‐weighted distance rasters produced in earlier steps. Primary linkages contained the Least Cost Paths. We also modeled secondary linkages, which are linkages that accrue low weights but are not the absolute lowest between two patches (McRae & Kavanagh, 2011). The value of the Least Cost Path was then subtracted from its surrounding linkage so that each primary linkage contained a Least Cost Path valued at zero with the surrounding pixels showing increasingly costly routes. For each Least Cost Path, we used Linkage Mapper to calculate the Euclidian distance between adjacent Habitat Concentration Areas, the cost‐weighted distance of each Least Cost Path, and the un‐weighted length of each Least Cost Path. We also calculated the cost‐weight accumulated along each path divided by the Euclidian distance. The accumulated cost‐weight along each path was divided by the un‐weighted length of the path, providing the average resistance a lynx would face while traveling along each Least Cost Path. Ratios closer to 1 represent higher quality paths (WWHCWG, 2010).

## 3 RESULTS

### 3.1 Habitat concentration areas and the resistance surface

**Figure 2:**
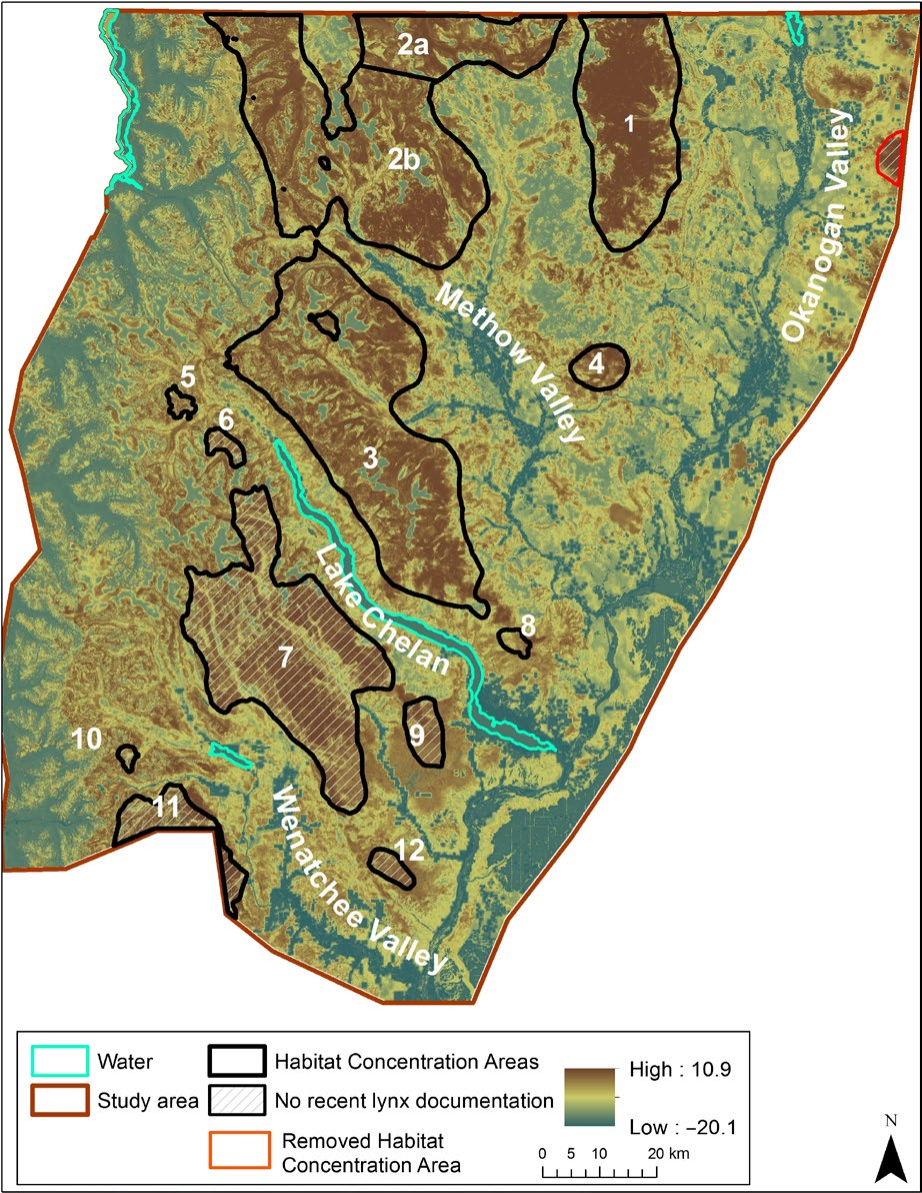
Habitat Concentration Areas identified within the North Cascades study area

**Figure 3:**
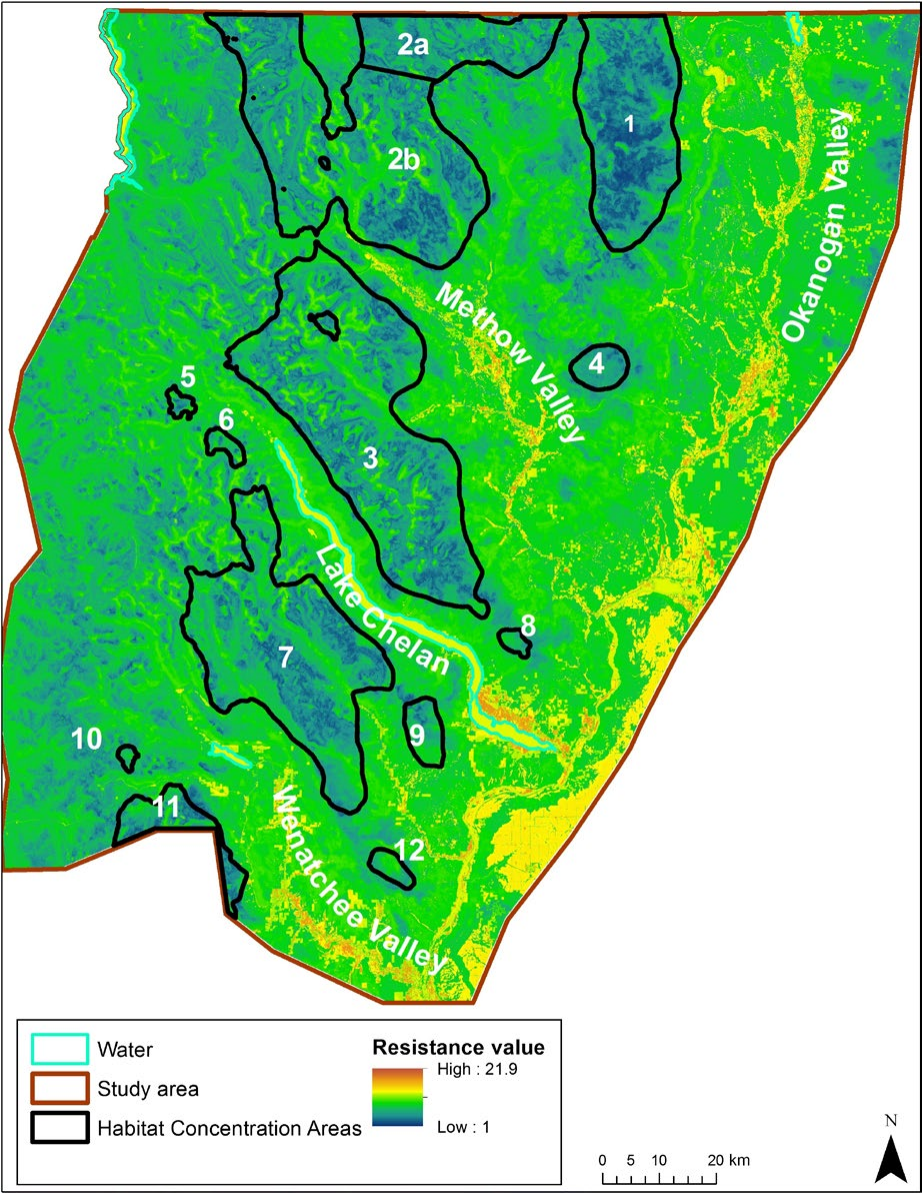
The resistance surface for lynx movement within the North Cascades

We identified 12 Habitat Concentration Areas ranging from 10 to 1,459 km^2^ (Table 3, Figure 2). The habitat quality raster for lynx in the North Cascade Mountains had values that ranged from −0.1 to 10.9 (mean: 2.2, *SD*: 3.3; Figure 2). Although the majority of each Habitat Concentration Area lies within the Okanogan Lynx Management Zone (Stinson, 2001), the southernmost Habitat Concentration Area (area 11) is south of Highway 2 and outside the Lynx Management Zone. Three Habitat Concentration Areas are smaller than the smallest home range identified for lynx in this study, but can still provide valuable patches of core habitat for lynx passing through an area. The final resistance surface values ranged from 1 to 21.9 (mean: 8.2, *SD:* 2.5; Figure 3).

**Table 3:** Habitat Concentration Areas identified for lynx in the North Cascades

Habitat concentration area	Area (km^2^)	Average habitat value	*SD* of habitat value
Lynx likely present			
2	1,459	4.6	1.7
3	1,272	4.5	1.5
1	599	5.9	2.4
4	60	4.5	1.4
**8**	**17**	**3.9**	**0.8**
Lynx probably absent			
7	926	4.6	1.5
11	126	4.6	1.6
9	64	4.5	1.0
12	30	4.5	0.7
6	24	4.1	1.2
**5** [Link]	**16**	**4.0**	**1.1**
**10**	**10**	**3.9**	**1.0**

Bold fonts indicate areas smaller than the smallest home range estimated for lynx in this study.

The radio‐collared male lynx, 312, went on several long forays. He visited this site in passing. We do not have other evidence of lynx in this area.

### 3.2 Connectivity models

**Figure 4:**
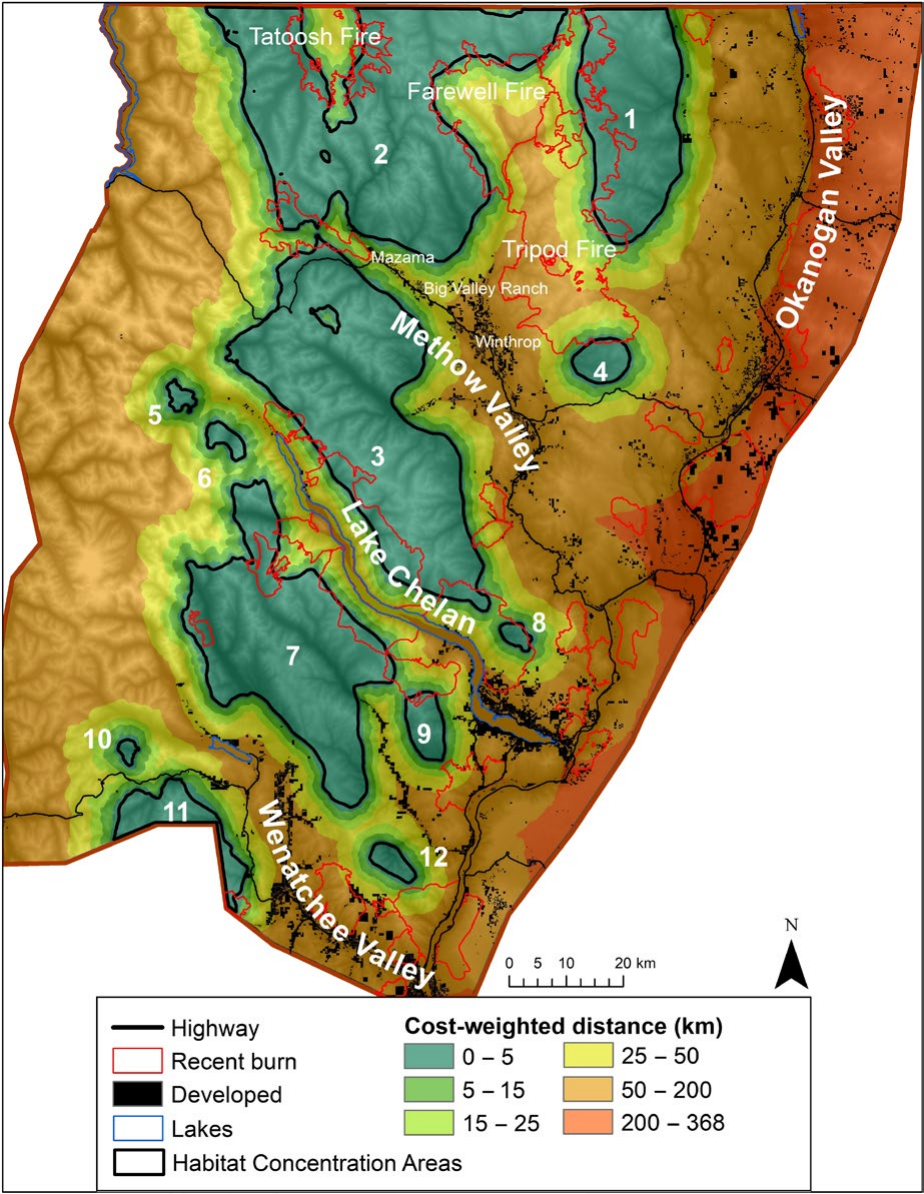
Cost‐weighted distance map symbolizing the difficulty for lynx of moving from any pixel to the nearest Habitat Concentration Area. Recent burns occurred between 1995 and 2012

The cost‐weighted distance map (Figure 4) highlights that cost is low for lynx moving in the sub‐boreal and mixed forest zone but quickly accumulates to the east of the mountains toward the low‐elevation Okanogan Valley and west of the Cascade crest where moister forests dominate. Weighted cost also increases in the Methow and Wenatchee Valleys and around Lake Chelan, all areas with more open and human‐dominated habitats. Within high‐elevation forested areas, burns such as the 2003 Farewell Fire and the 2006 Tatoosh and Tripod Fires increased resistance, but fire “skips” (unburned patches within the fire perimeter) and regenerating forest lower the resistance to lynx movement through these areas.

**Figure 5:**
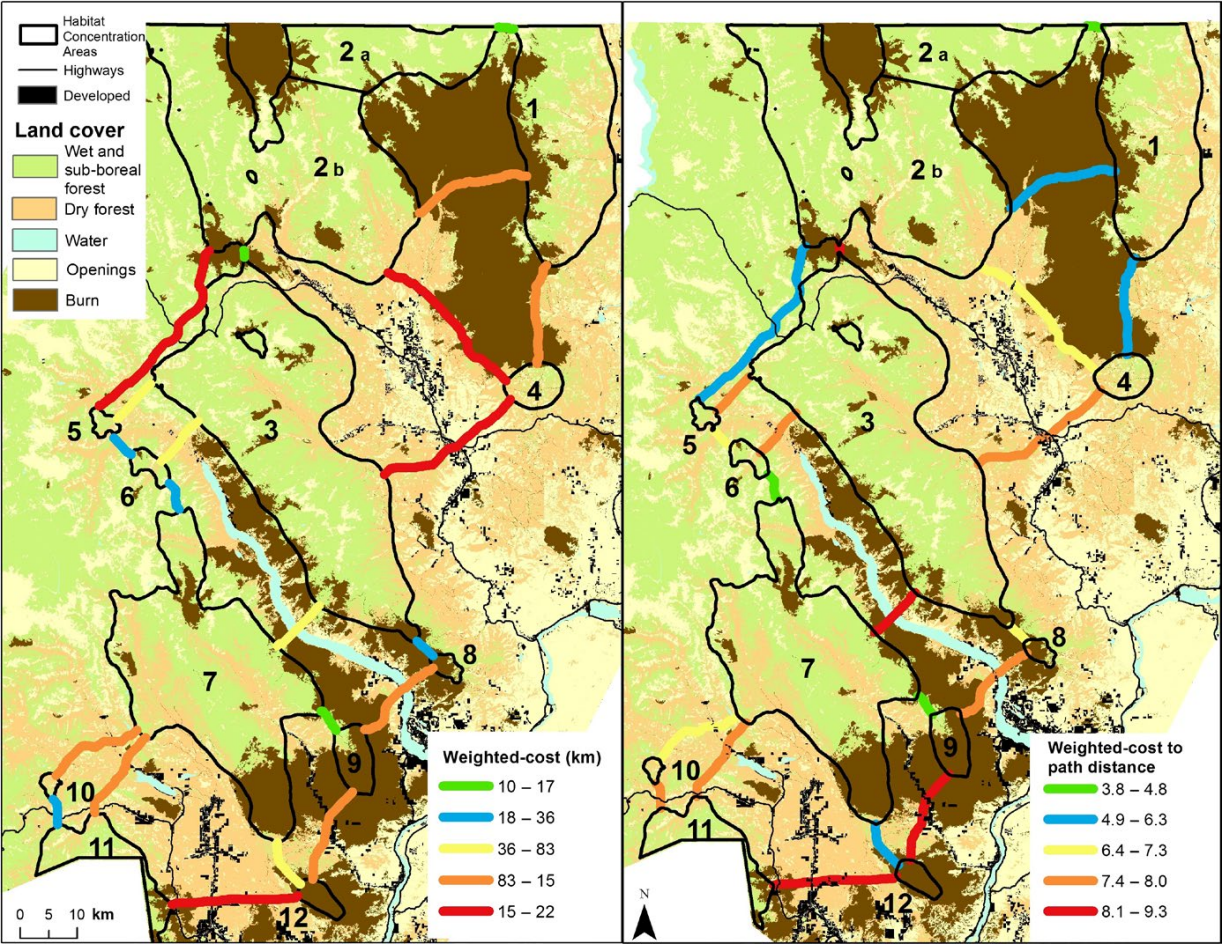
Least Cost Paths connecting Habitat Concentration Areas in the North Cascades. The total weighted cost of each Least Cost Path (Map A) represents the accumulated resistance value of each path. The weighted cost to path distance of each Least Cost Paths (Map B) represents the accumulated resistance divided by the total un‐weighted distance of each path

**Figure 6:**
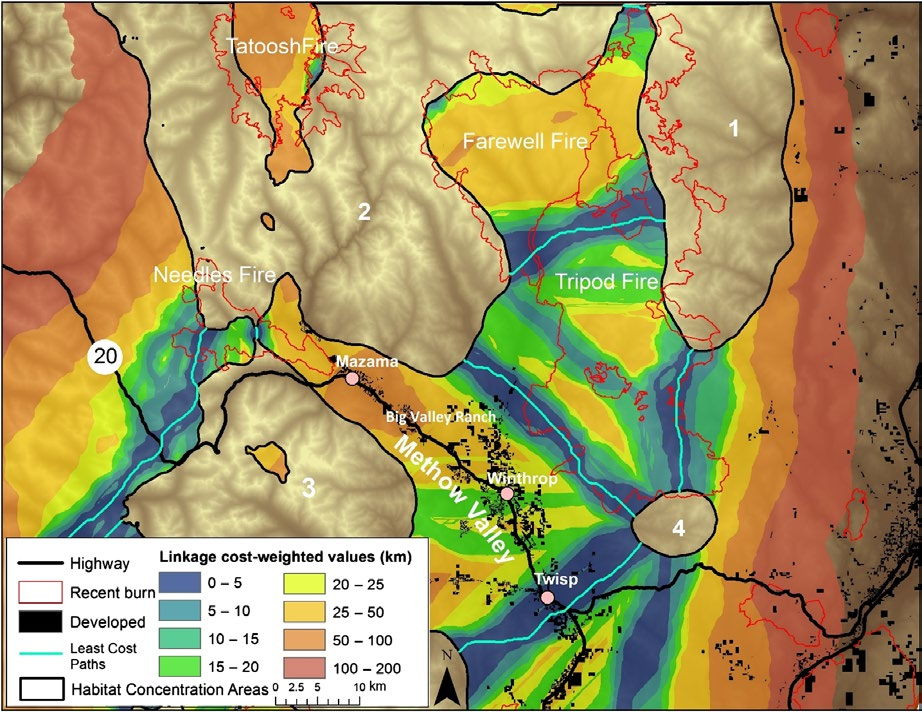
Linkages connecting Habitat Concentration Areas 1–5. The linkage map is scaled so that the Least Cost Path in a linkage equals zero with the alternative routes increasing in resistance as they emanate outward from the Least Cost Path. Thus, cool colors present the lowest resistance within that linkage to lynx movement while warmer colors in the linkage present higher resistance to movement. Because of this scaling, primary linkages cannot be compared to each other based on their color. Secondary linkages are scaled relative to the surrounding landscape and can be compared to each other based on their color

In some cases, more than one linkage between adjacent Habitat Concentration Areas was identified. To assist with identifying the primary linkage, Linkage Mapper also modeled the Least Cost Path between each pair of adjacent Habitat Concentration Areas, and identified 21 Least Cost Paths connecting the Habitat Concentration Areas into a single network (Figures 5 and 6). Each of the 21 Least Cost Paths had un‐weighted and weighted lengths shorter than 367 km, which was the longest dispersal distance by radio‐collared lynx in this study (Table 4, Figure 5). Cost‐weighted distances ranged from 10 to 215 km and weighted cost/path length ratios ranged from 4.8 to 9.3. Several paths stand out as connecting Habitat Concentration Areas with low accumulations of resistance (cost‐weighted distance) or low cost‐weight to path length ratios. For example, Least Cost Paths from Habitat Concentration Areas 2b and 3 to areas 5 and 6 represent high quality linkages that connect currently known lynx populations to Habitat Concentration Areas south of Lake Chelan where lynx are not currently known, but have been documented and could potentially recolonize (Table 4, Figure 5).

**Table 4:** Linkage statistics for evaluating the quality of each Least Cost Path for lynx in the North Cascades

Least cost path	Cost‐ weighted distance (km)	Euclidian distance (km)	Least cost path length (km)	Weighted cost divided by Euclidian distance	Weighted cost divided by path length
7–9	17	4	5	4.5	3.8
1–2a	14	3	3	5.3	4.8
6–7	25	4	5	6.0	4.8
2b−5	208	33	37	6.4	5.6
1–2b	127	18	21	7.0	5.9
1–4	111	17	18	6.7	6.1
7–12	56	8	9	7.4	6.3
5–6	29	4	4	7.1	6.7
4–2b	203	27	30	7.4	6.9
7–10	126	16	18	8.0	7.1
3–8	28	4	4	7.5	7.3
3–6	73	9	10	8.0	7.4
7–11	123	15	17	8.3	7.4
10–11	36	4	5	8.2	7.7
8–9	134	15	17	8.7	7.7
3–4	215	25	27	8.6	7.9
3–5	67	8	8	8.2	8.0
3–7	83	10	10	8.4	8.1
9–12	146	16	18	8.9	8.2
2b−3	10	1	1	8.8	8.3
11–12	208	22	22	9.6	9.3

Note

Lower costs indicate better connectivity.

## 4 DISCUSSION

Lynx are relatively specialized when it comes to selecting core habitat for hunting and resting, but lynx also travel long distances and through a variety of habitats generally not selected as core habitat and thus often labeled as matrix habitat (Mowat, Poole, & O'Donoghue, 2000; Squires & Laurion, 2000). Indeed, some of the GPS‐collared lynx in this study went on exploratory movements outside of their home ranges or dispersed into British Columbia, traversing high peaks above tree line and recently burned areas. These lynx also crossed valley bottoms with farmland and human development, open sage or grass lands, and over several highways (Supporting information Figure S1). However, lynx’ ability to travel through a variety of habitats is not as contradictory as it may seem to their more particular core habitat selection. Our models show that within matrix areas lynx select for certain characteristics so that our connectivity models showed some areas of the matrix as providing poor connectivity and others as providing much better connectivity. Core lynx habitat is forested (Koehler et al., 2008; Maletzke, Koehler, Wielgus, Aubry, & Evans, 2008; Squires, Decesare, Kolbe, & Ruggiero, 2010) and similarly, lynx prefer to travel through matrix areas that provide some amount of cover. Areas without forest cover, such as open sage‐steppe and human‐dominated areas, are less desirable to traveling lynx.

Several other recent studies on lynx have also highlighted how lynx navigate in complex landscapes. Farrell et al. (2018) examined lynx connectivity in the northeastern US, finding that lynx strongly prefer areas with natural forest cover. Holbrook, Squires, Olson, DeCesare, and Lawrence (2017) examined lynx in the northern US Rockies, focusing on identifying where home ranges were located (mature conifer forests were preferred) and use of habitats within home ranges. Akin to our results, they showed lynx routinely cross areas of less suitable habitat to spend more time in preferred habitats. Buderman, Hooten, Ivan, and Shenk (2018) document movements of lynx that were reintroduced to Colorado, finding that most animals explored a number of locations and crossed a wide variety of habitat types before settling into home ranges. They documented lynx traveling through habitats that would not be identified as core or high quality lynx habitat. These studies focused on habitats lynx prefer; our models therefore differ because we explicitly based our connectivity models on habitats lynx do not prefer but are still willing to use. Our results suggest that lynx connectivity may be higher than reported by these other studies, simply because the other models may have missed suitable linkages that are not good lynx habitat but that are capable of supporting dispersal. We also note that these models from different regions pick up different individual habitat variables as important to lynx, reinforcing the value of developing models from local data when possible.

We identified twelve Habitat Concentration Areas in the North Cascades. Although the six areas south of Lake Chelan (5–7 and 9–12) are not currently known to support resident lynx and their most recent lynx documentation was in 1991 (Stinson, 2001; R. Naney, personal communication), these Habitat Concentration Areas are within the historical range of lynx and could conceivably be occupied in the future, especially during peaks of the snowshoe hare cycles when lynx numbers are high and populations can expand (Schwartz, Mills, McKelvey, Ruggiero, & Allendorf, 2002). The modeled Habitat Concentration Areas do not represent all lynx habitat in the North Cascades; core lynx habitat of lower but suitable value exists outside of the Habitat Concentration Areas. Three Habitat Concentration Areas were <19 km^2^, which is the smallest home range size identified for a lynx in this study. While these small Habitat Concentration Areas may not be large enough to support a lynx, they can act as “stepping stones” (Dickson, Roemer, McRae, & Rundall, 2013) for lynx to hunt in while passing through an area. Alternatively, since these small Habitat Concentration Areas are surrounded by lower quality but still core habitat, they may indeed indicate broader areas capable of supporting lynx.

The cost‐weighted map depicts the overall matrix permeability (Figure 4) and is perhaps the most informative and important product of this connectivity analysis (WWHCWG, 2010). Although it does not specifically highlight linkages, the cost‐weighted map contains linkage information since the linkage map is simply the sum of individual, adjacent cost‐weighted maps (WWHCWG, 2010). In addition, this map portrays the full range of areas a traveling lynx may use and allows easy comparison of the qualities of different linkage areas. For example, the northern end of the Tripod burn supported high connectivity between Habitat Concentration Areas 1 and 2. Finally, the cost‐weighted distance map highlights broad areas of low resistance and broad areas of high weighted cost where connectivity is low or in need of restoration (Figure 4).

The cost‐weighted distance map also illustrates the value of basing resistance surfaces on models of matrix habitat selection. For example, the area between Habitat Concentration Areas 2 and 3 (crossing Route 20 between Big Valley Ranch and Mazama) showed up as quite permeable to lynx, despite the area having no sub‐boreal forest, relatively open habitats, and lower elevations. Had we built our resistance surfaces from only our Core Habitat Model, it is unlikely this area would have been identified for connecting lynx habitats.

The linkage map also highlights where primary linkages (those that contain a Least Cost Path) and secondary linkages exist between Habitat Concentration Areas. Least Cost Paths themselves are only one‐pixel‐wide pathways and are therefore sensitive to errors in the underlying GIS layers used to create the resistance surface, as well as our knowledge about the habitat suitability for the species of interest. Least Cost Paths themselves therefore should not be interpreted or used as an exact map of a linkage. Instead, focusing on the alternative routes clustered around the Least Cost Path indicates a broader area of low resistance. Several such linkages clearly emerge at the northern end of the study area (Figure 6). Again, basing our analysis on the Matrix Habitat Model was useful because primary and secondary linkages were identified across the recent Tripod burn. Although such habitats are rarely used by lynx, the Matrix Habitat Model clearly identified that lynx could use them. Had we just used our Core Habitat Model, which shows little use of burns by lynx (Vanbianchi, Murphy, & Hodges, 2017), these linkages would not be detected. Indeed, we observed male lynx 312 crossing Tripod burn in 2012, just 6 years after the fire, using a route near a modeled secondary linkage (Supporting information Figure S1).

One disadvantage of the linkage map compared to the cost‐weighted map is that the linkage map can give the false impression that suitable habitat for traveling is limited to the best primary and secondary linkage areas. For example, the Mazama and Big Valley Ranch areas are identified by the cost‐weighted map as having fairly high connectivity between Habitat Concentration Areas 2 and 3. However, in the linkage map, this same area is portrayed as having low connectivity because it is scaled relative to the Least Cost Path connecting Habitat Concentration Areas 2 and 3. Indeed, one lynx radio‐collared for this study (male 312) crossed the Methow Valley near Mazama, demonstrating that in addition to modeled linkages, low resistance areas identified by the cost‐weighted map are important to connectivity (Figure 4, Figure 6; Supporting information Figure S2).

Once linkages are identified by the cost‐weighted and linkage maps, these areas must be evaluated since their presence does not guarantee that they are suitable for lynx to travel through, only that they are zones of low resistance between Habitat Concentration Areas. For example, the linkage connecting Habitat Concentration Areas 3 and 4 may be the best available route between those areas, but the linkage is poor since it passes through developed and open areas (Figure 6). Conversely, the linkages connecting areas 2b and 4 and areas 1 and 4 are more suitable since they traverse forested areas away from human development (Figure 6).

**Figure 7:**
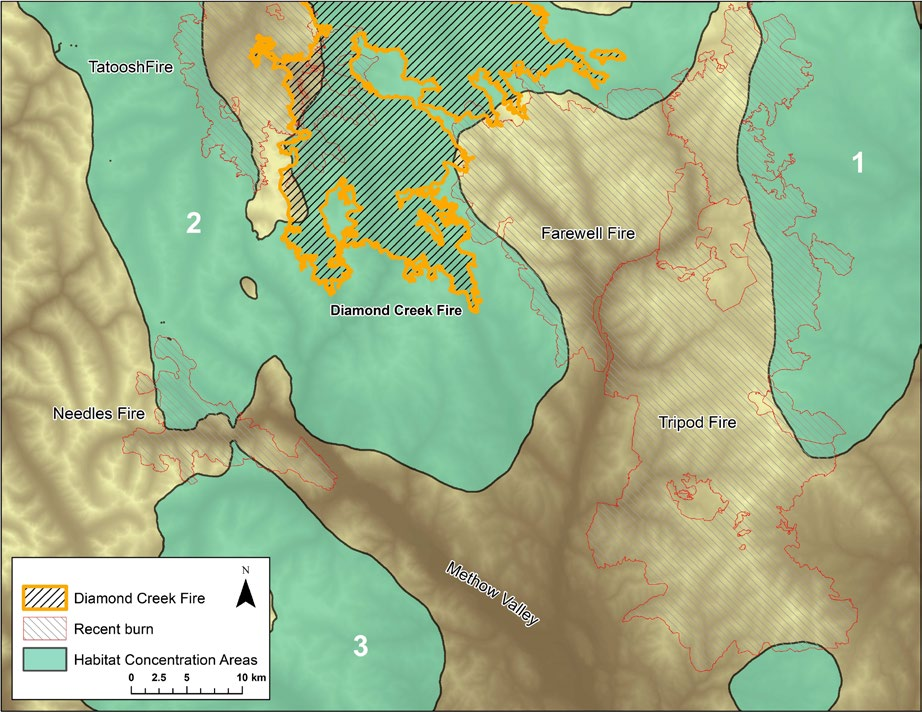
The 2017 Diamond Creek Fire burned a significant portion of high quality lynx habitat in the largest lynx‐occupied Habitat Concentration Area (2) in Washington. The Diamond Creek Fire perimeter extends into British Columbia; shown here is Washington only

To create these connectivity models, we used the best available GIS layers, current to ~2012. However, spatial connectivity models are sensitive to the quality and scale (spatial and temporal), of the underlying data. Human development and natural impacts such as fire will continue to change lynx habitat connectivity within the North Cascades ecoregion. Indeed, since this analysis was completed, the 2017 Diamond Creek Fire burned more than 51,648 ha of forest in the heart of North Cascades lynx habitat with 39,326 ha of the fire burning in Washington and 12,322 ha in British Columbia. The fire burned within Habitat Concentration Area 2, which was the largest lynx‐occupied Habitat Concentration Area in Washington; the fire impacted ~24% of Habitat Concentration Area 2, an area the size of 4.5 average‐sized lynx home ranges (Figure 7). The spatial arraignment of the Diamond Creek Fire may present additional impacts to North Cascades lynx; the fire burned between the Tripod, Farewell, and Tatoosh fire scars, all recent fires that have not yet regenerated into quality lynx habitat and present higher resistance to lynx movement. With the Diamond Creek Fire scar now filling the space between the Tripod, Farewell, and Tatoosh burns, a contiguous swath of high resistance burn area now runs southeast through the Cascades, leaving only the areas along the west and east edges of North Cascades lynx habitat well connected to habitat further north in BC. Finally, the meso‐landscape scale at which we modeled linkages does not imply that important linkages do not exist within smaller areas, simply that these models were not created at that resolution. Modeling linkages at a large scale is an area for further study that could build upon and complement the work done in this study.

### 4.1 Implications for management and conservation

Lynx in the North Cascades must move across the landscape to disperse, explore, find mates, and escape habitat degradation after disturbances such as fire. New burns reduce forest cover and thus reduce connectivity for lynx. Residual forest structures, especially in fire skips, provide valuable cover for lynx crossing recent burns. For this reason, retaining residual structure post‐burn will provide cover for lynx and also promote growing conditions for regenerating vegetation, allowing burned areas to recover more quickly (Brassard & Chen, 2006). Human‐populated valley bottoms also create areas of higher resistance to lynx movement. Linkages across valley bottoms are also more vulnerable since expanding human developments degrade connectivity. Areas of connectivity identified in these models across open and developed valley bottoms provide direction for conducting field‐based assessments and validation of linkages so that managers can prioritize and conserve these vulnerable linkages.

In a landscape continually impacted by a growing human presence and increasing wildfires, identifying and conserving areas that facilitate lynx movement will help to ensure that dispersing lynx reach new home ranges, find mates, escape degraded habitats, and exchange genes. This study is the first model of meso‐scale connectivity in the North Cascades to be built using animal GPS data and, importantly, incorporates lynx response to burned areas, an aspect of lynx habitat use that has previously received little attention. These models provide an overview of core lynx habitat and where important linkages may exist, lending land managers a guide for focusing future work that validates and prioritizes lynx habitat linkages in the North Cascades.

Our approach also clearly highlights the value of building separate habitat use models for animals within their core habitats and for animals traveling between resource patches or dispersing. Quite simply, traveling animals tolerate poorer habitats, which means landscape permeability is likely higher than is modeled when researchers build habitat models focused on core habitat selection and from locations pooled across an animals home ranges. In our case, lynx clearly still preferred the same *kinds* of features (especially forest cover) when traveling, but essentially lowered their standards. This finding corroborates other recent studies’ findings that for kinkajous (*Potos flavus*) in Central America, elk (*Cervus canadensis*), bighorn sheep (*Ovis canadensis nelson*), and red‐cockaded woodpeckers (*Picoides borealis*) in North America, and Eurasian brown bears (*Ursus arctos*) and Iberian lynx (*Lynx pardinus*) in Europe, core habitat selection does not reflect the full spectrum of habitat selection during movements outside the home range. For each of these species, specific core habitat needs were relaxed to accept lower‐quality habitats while animals were moving across the landscape (Blazquez‐Cabrera et al., 2016; Keeley et al., 2016, 2017 ; Mateo‐Sanchez et al., 2015; Trainor et al., 2013). As this building mass of evidence indicates, it is important that connectivity conservation move away from a narrow focus on protecting structural habitat corridors, and toward functional connectivity and maintaining landscapes that are more broadly permeable because of the range of cover types that traveling animals can use. Maintaining such poor‐but‐useful habitats may become especially critical as severe wildfires become increasingly common and forest wildlife need to move between remnant patches of core habitat as recently burned areas regrow into more suitable conditions.

## CONFLICT OF INTEREST

None declared.

## AUTHOR CONTRIBUTIONS

CV and KEH conceived the ideas and approach and obtained permission to use the data; WLG and MAM helped develop GIS layers and RF models; CV led the data analysis; CV and KH led the writing of the manuscript. All authors contributed critically to the drafts and gave final approval for publication.

## DATA ACCESSIBILITY

Our work made use of radio‐collared lynx data, but this radio‐collaring was undertaken by government agencies rather than under the auspices of the university.  The data have been archived with the relevant federal and state agencies, including US Fish and Wildlife Service, US Forest Service, and the Washington Department of Fish and Wildlife.
