# Supplementary figures and images for: Navigating fragmented landscapes: Canada lynx brave poor quality habitats while traveling
Source: Ecol Evol. 2018 Oct 26;8(22):11293–308. doi: 10.1002/ece3.4605 (PMC6262728; doi:10.1002/ece3.4605)

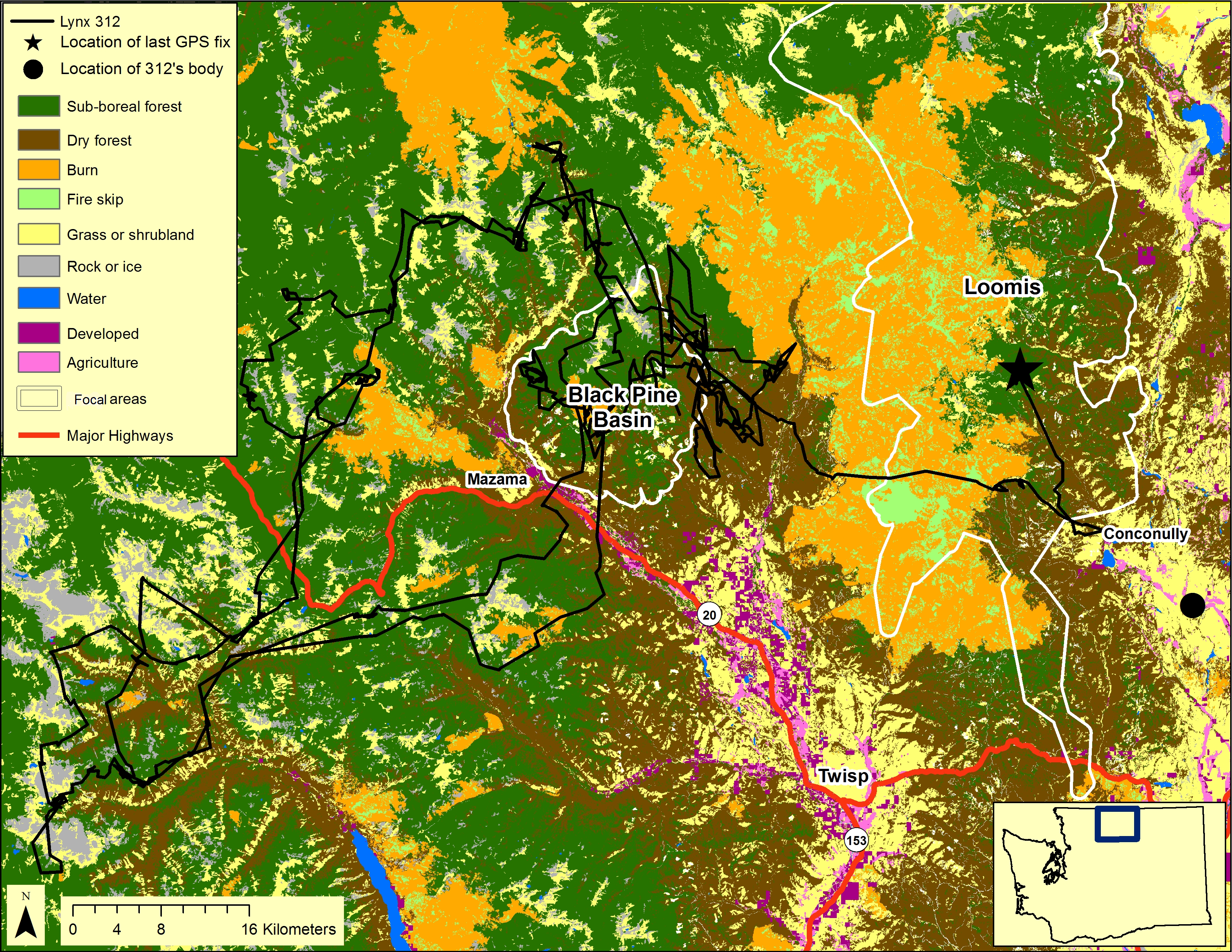

Supplement: Supplementary file 1 [file ECE3-8-11293-s001.tif]

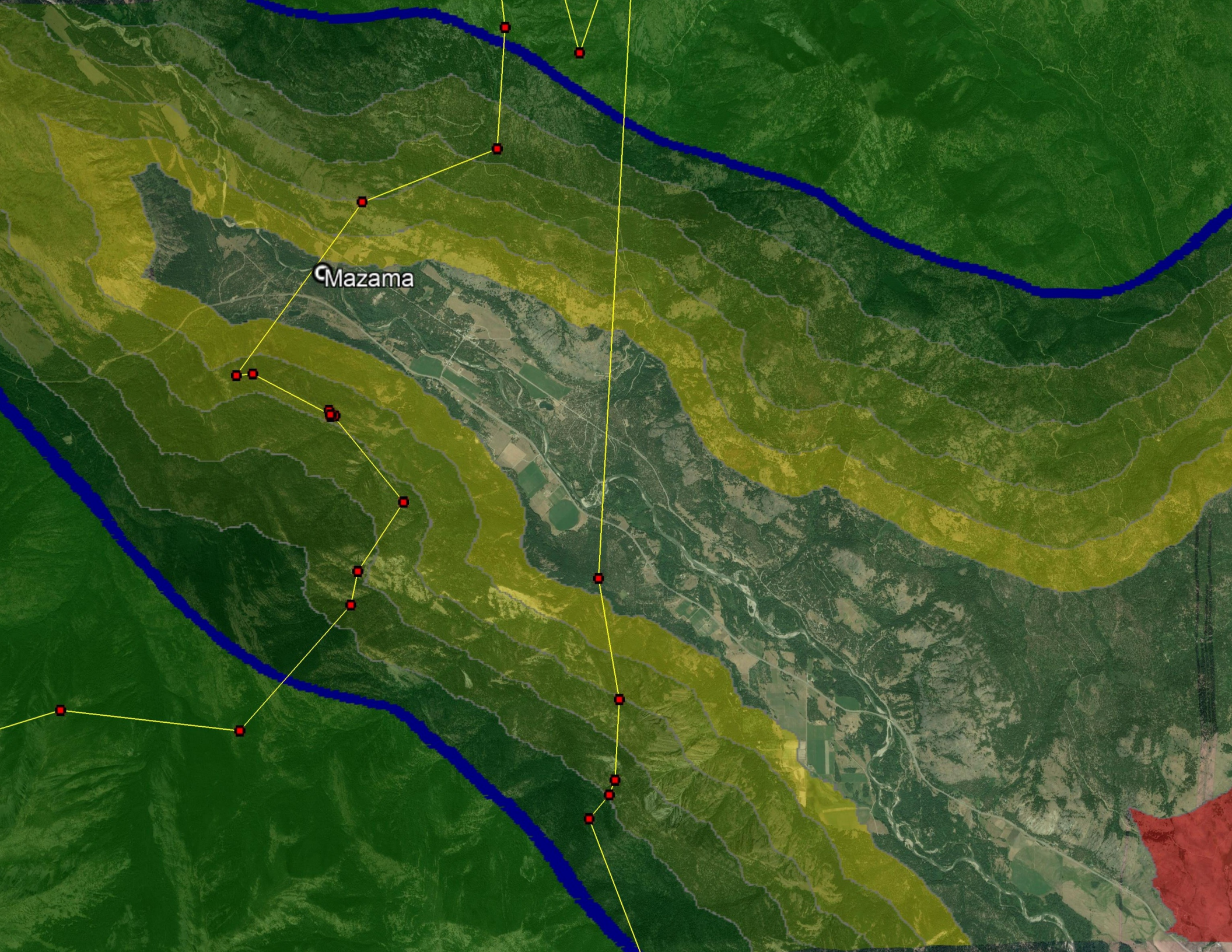

Mazama

Supplement: Supplementary file 2 [file ECE3-8-11293-s002.pdf]
