# Supplementary material for: Navigating fragmented landscapes: Canada lynx brave poor quality habitats while traveling
Source: Ecol Evol. 2018 Oct 26;8(22):11293–308. doi: 10.1002/ece3.4605 (PMC6262728; doi:10.1002/ece3.4605)
Supplement: Supplementary file 3 [file ECE3-8-11293-s003.docx]

Supporting Material

**Figure S1.** Male 312’s movement pathway in the North Cascades.  His GPS collar stopped collecting locations while he was within the Loomis focal area.  His body was found outside of the Loomis focal area near Conconully, Washington.  312’s travel path clearly demonstrates the use of travel habitat types such as open areas and developed areas that are not selected as core habitat.

**Figure S2.** Male 312’s movement pathway across the Methow Valley overlaid onto a Google Earth image (Google Earth 2018).  Blue lines delineate Habitat Concentration Areas 2b (top) and 3 (bottom).  Shading from red (high) to dark green (low) represent cost-weighted distance as it radiates away from the closest Habitat Concentration Area.  The yellow line is the straight line distance between 312’s GPS locations, represented by red dots. This figure exemplifies a lynx’ movement path that crosses poor habitat (dry forest, human development, and Highway 20) but still offers low resistance.  Here, 312 crossed the developed Methow Valley bottom near Mazama in an area identified in our model as being an area of low cost-weighted distance.
